# Stress and depression among students: Are we doing enough?

**DOI:** 10.12669/pjms.38.8.7076

**Published:** 2022

**Authors:** Sundus Tariq, Saba Tariq, Naeem Mubarak

**Affiliations:** 1 Sundus Tariq, Professor Physiology, University Medical & Dental College, The University of Faisalabad, Pakistan, University of Health Sciences, Lahore, Pakistan; 2 Saba Tariq, Professor Pharmacology and Therapeutics, University Medical & Dental College, The University of Faisalabad, Pakistan, University of Health Sciences, Lahore, Pakistan; 3 Naeem Mubarak Associate Professor, Basic Medical Science Department, Lahore Medical & Dental College, University of Health Sciences, Lahore, Pakistan

A researcher unable to meet the deadlines or facing repeated failures in publishing a manuscript, a student worried about upcoming exams and future, a young scholar coming unprepared in tutorial or a meeting with the fear of getting laid off or making a mistake, an introvert who has to face a large audience for a presentation, or someone who is suffering from a disease or fear of losing a loved one. These are a few of the stressors that almost every student comes across. Stress is defined as a state of homeodynamic imbalance,^1^ which is normal part of life.

Short-term stress initiates a fight and flight response, that is beneficial and a natural mechanism to cope up with the challenges of daily life by enhancing the physiological and psychological processes of human body.^2^ It is imperative that these altered physiological and psychological changes return to baseline before the next stressor hits, in order to retain the optimal functionality of human body. In the presence of chronic stress, the stress related biological changes failed to resolve back to the baseline within the time frame leading to deleterious effects on both physical and psychological health.^2^

A person suffering from chronic stress can have behavioral changes, attention deficit, altered sleep and eating patterns, memory impairment, frequent headaches and inability to accomplish a task. It can further lead to anxiety and depression if not relieved timely. More than 40% of the undergraduate students were suffering from stress in a single center study and the major stressors turned out to be raised parental expectations, uncertainty about their future, accommodation away from home and frequent examinations.^3^ Two independent systematic reviews revealed that depression prevailed among 27%^4^ to 42.66%^5^ of the undergraduate students in Pakistan. The reasons were again high parental and societal expectations and limited opportunities in highly competitive job market.^5^ Interestingly in a study, stress was also linked to unhealthy eating habits of the students especially in males.^6^

In the past two years, COVID-19 has caused interference in normal life of every individual. The impact of COVID-19 on student’s mental health as well as lifestyle further adds to stress. A few studies conducted on medical students in Pakistan, unraveled the hidden crises related to this pandemic and its damaging effects on mental health and lifestyle of these students.^7^,^8^ In one study, more than 48% of the students suffered from depression and anxiety. Alarming finding was that one in five thought that it would be better if they were dead, and 8% admitted to often think of committing suicide during the past two weeks.^7^ Another study showed that more than 50% of the medical students reported increase in screen time, weight gain and poor sleep while increasing their use of sleeping pills (3.1%). On the other hand, exercise and increasing the physical activity helped in reducing their anxiety.^8^

Considering the high prevalence of stress and depression in Pakistani students, it is vital that the stressors should be dealt with at an early stage and students should be taught coping strategies to overcome them. If this is not undertaken at institutional levels, the students cannot contribute effectively in their personal as well as professional lives or the healthy psychological and physical development and a smooth transition to their adulthood, it is essential that everyone, including their peers, family members, friends, faculty, institutional heads and policy makers play their part effectively.

In this regard, adopting a policy “To put people first; listen first” could be a great start. Recruitment of qualified counselors and mental health educators at university level educations should be a binding.

Some institutes do have student counseling cells with a trained psychologist to guide the students in managing their thoughts and stress. But this is not enough; stress management should be a part of the curriculum, faculty should be trained to identify and deal with common psychological issues. World health organization has created an illustrated guide “Doing what matters in time of stress” for public use for stress management.^9^ This guide beautifully illustrates the five steps that can be adopted to cope with adversity. The guide is based on the concepts of grounding yourself, unhooking yourself, acting on your values, being kind that goes both ways and engaging with the world around you after you make room for your painful feelings or difficult though processes. By doing so you can create more satisfying and fulfilling life. Furthermore, World Health Organization recommends “Talk Therapy” as the first line of treatment for mild-to-moderate depression and anxiety. This can be delegated to mental health educators. Additionally, availability of self-assessment mental health tools, periodic screening camps and brochures or flyers to enhance the awareness on the issue should be arranged from time to time.

The World Psychiatric Association working group on medical students has developed a module on medical student’s wellbeing which has also been adopted by faculty in Pakistan, Canada and medical students themselves. It is available free of cost for students all over the globe to help students learn about stress and its management.^10^,^11^

It is not possible to always avoid stressors in life, but there are various ways by which we can optimize the good stress and minimize bad/ chronic stress.^2^ Modifying life style factors like sleep, nutrition and physical activity will help regulate the stress levels. The addition of psychosocial buffers like use of coping strategies, appraisal, social support, compassion, gratitude will definitely minimize stress and doing activities like meditation, exercise, laughing, making new friends, learning something new, helping others or volunteering for community work, avoiding alcohol and drugs, indulging in nature, art, craft and painting are few of the other ways that can help an individual to get rid of stress and function in a better way.

Stress, especially when chronic, is a serious issue, and through this editorial, we are trying to make visible a hidden crisis ahead of the time in the conservative society of Pakistan. Taking care of mental health of students is associated with better physical health outcomes, more engaged students and lower failure rates. Educational institutes cannot afford to ignore this dimension of well-being, thus, building a positive mental health culture in educational institution is inevitable.
